# Pediatric diffuse intrinsic pontine glioma radiotherapy response prediction: MRI morphology and T2 intensity-based quantitative analyses

**DOI:** 10.1007/s00330-024-10855-9

**Published:** 2024-06-21

**Authors:** Xiaojun Yu, Shaoqun Li, Wenfeng Mai, Xiaoyu Hua, Mengnan Sun, Mingyao Lai, Dong Zhang, Zeyu Xiao, Lichao Wang, Changzheng Shi, Liangping Luo, Linbo Cai

**Affiliations:** 1 Department of Medical Imaging Center, Jinan University First Affiliated Hospital, No. 613, Huangpu Road West, Tianhe District, Guangzhou, 510630 Guangdong Province China; 2 Department of Oncology, Guangdong sanjiu Brain Hospital, No. 578, Shatai South Road, Baiyun District, Guangzhou, 510510 Guangdong Province China

**Keywords:** Diffuse intrinsic pontine glioma, Magnetic resonance imaging, Child, Radiotherapy

## Abstract

**Objectives:**

An easy-to-implement MRI model for predicting partial response (PR) postradiotherapy for diffuse intrinsic pontine glioma (DIPG) is lacking. Utilizing quantitative T2 signal intensity and introducing a visual evaluation method based on T2 signal intensity heterogeneity, and compared MRI radiomic models for predicting radiotherapy response in pediatric patients with DIPG.

**Methods:**

We retrospectively included patients with brainstem gliomas aged ≤ 18 years admitted between July 2011 and March 2023. Applying Response Assessment in Pediatric Neuro-Oncology criteria, we categorized patients into PR and non-PR groups. For qualitative analysis, tumor heterogeneity vision was classified into four grades based on T2-weighted images. Quantitative analysis included the relative T2 signal intensity ratio (rT2SR), extra pons volume ratio, and tumor ring-enhancement volume. Radiomic features were extracted from T2-weighted and T1-enhanced images of volumes of interest. Univariate analysis was used to identify independent variables related to PR. Multivariate logistic regression was performed using significant variables (*p* < 0.05) from univariate analysis.

**Results:**

Of 140 patients (training *n* = 109, and test *n* = 31), 64 (45.7%) achieved PR. The AUC of the predictive model with extrapontine volume ratio, rT2SRmax–min (rT2SR_dif_), and grade was 0.89. The AUCs of the T2-weighted and T1WI-enhanced models with radiomic signatures were 0.84 and 0.81, respectively. For the 31 DIPG test sets, the AUCs were 0.91, 0.83, and 0.81, for the models incorporating the quantitative features, radiomic model (T2-weighted images, and T1W1-enhanced images), respectively.

**Conclusion:**

Combining T2-weighted quantification with qualitative and extrapontine volume ratios reliably predicted pediatric DIPG radiotherapy response.

**Clinical relevance statement:**

Combining T2-weighted quantification with qualitative and extrapontine volume ratios can accurately predict diffuse intrinsic pontine glioma (DIPG) radiotherapy response, which may facilitate personalized treatment and prognostic assessment for patients with DIPG.

**Key Points:**

- *Early identification is crucial for radiotherapy response and risk stratification in diffuse intrinsic pontine glioma.*
- *The model using tumor heterogeneity and quantitative T2 signal metrics achieved an AUC of 0.91.*
- *Using a combination of parameters can effectively predict radiotherapy response in this population.*

## Introduction

Diffuse intrinsic pontine glioma (DIPG) is an aggressive malignant childhood brainstem tumor with a median overall survival of less than 1 year [1, 2]. The location of these tumors in an eloquent brain region and their infiltrative growth pattern preclude surgical resection. Radiation therapy, the standard of care [3], can prolong the survival by 3- to 4-months [4, 5]; however, chemotherapy does not prolong the survival in patients with DIPG. Although our understanding of DIPG continues to expand, the diagnosis is based on characteristic radiological and clinical features without routine histopathological corroboration. Recently, mutations in genes encoding histones H3F3A and HIST1H3B have been found to be the main feature of DIPG, and DIPG has been rated as a grade IV brain tumor by WHO because of highly aggressive behavior and poor prognosis [6]; In the 2021 WHO classification, diffuse midline gliomas H3 K27M-mutant were renamed to diffuse midline gliomas H3 K27-altered [7].

The baseline imaging features associated with worse overall survival (OS) include large size, necrosis, ring enhancement, diffusion restriction, extension of extrapontine lesions, and distant disease [1, 8, 9]. However, these data have not helped in making initial treatment decisions for DIPG. Patients with better responses to radiotherapy have longer OS [9–11]. Further, the importance of biopsy and thorough molecular research for some patients with radioresistant DIPG have been emphasized. Provide potential possibilities for targeted therapy or intratumoral infusion administration to achieve survival benefits for patients with radioresistant DIPG [12–15]. DIPG patients with better responses to radiotherapy may be a potential candidate to be treated with increased effective biological dose of radiotherapy [16]. With the increasing frequency of reirradiation in DIPG at first progression [17], patients with an initial radiotherapy response are most likely to benefit from it [18]. Therefore, early identification of the radiotherapy response to DIPG and clinical risk stratification are crucial.

Patients with wild-type TP53 (TP53^WT^) respond well to radiation therapy [18]. However, this hypothesis has not yet been confirmed. One study showed that patients with the T2-fluid-attenuated inversion recovery (FLAIR) mismatch sign showed good radiation response [16]. However, the cited study also had some limitations, including the small number of patients from whom clear conclusions could not be drawn and the lack of magnetic resonance (MR) image heterogeneity analysis. Furthermore, T2-weighted images were superior to other MR image types in determining DIPG intratumoral heterogeneity [19].

Therefore, this study aimed to use quantitative T2 signal intensity to develop a visual evaluation method based on T2 signal intensity heterogeneity and compare the use of MRI (T2-weighted and T1WI-enhanced) radiomic models to predict radiotherapy response in pediatric patients with DIPG.

## Materials and methods

The institutional review committee of our center approved this retrospective study and waived the requirement for informed consent.

### Study patients

We retrospectively included patients with brainstem gliomas aged ≤ 18 years who were admitted between July 2011 and March 2023. The exclusion criteria were as follows: (a) Less than 50% pontine involvement; (b) WHO grade 1 glioma, (c) Incomplete conventional radiation therapy at a dose of 54 Gray (Gy); (d) Unavailable or incomplete baseline or post-radiotherapy information. All the patients received a conventional radiation dose of 54 Gy in 30 fractions. For independent validation, patients admitted before October 2021 were assigned to the training set, and subsequent patients were assigned to the testing set.

All patients underwent brain MRI in the 3 weeks before and 4–6 weeks after the radiotherapy. OS was defined as the time from the date of diagnosis to the date of death.

### MRI parameters

MR images were obtained using a 3.0-T or 1.5-T MR scanner. All MRI examinations included axial T2WI, T2-FLAIR, T1WI, sagittal T1WI, and contrast-enhanced T1WI. Details are provided in Supplementary Table 1.

### Clinical variables

Clinical information on sex, age, DIPG diagnosis time, and Karnofsky performance status at diagnosis and after radiotherapy, was obtained from the patients’ medical records.

### Neuroimaging Analysis

All MR scans were reviewed by three observers (with 15, 7, and 9 years of radiology imaging experience), who were blinded to the patient’s history. The mean of the observer’s measurements was used as the final value for each quantitative parameter. Qualitative parameters were first independently reviewed by two observers (with seven and nine years of radiology imaging experience), who were blinded to the results of the other observers. If there were discrepancies in the results, another observer with 15 years of radiology imaging experience made the judgment.

**Fig. 1:**
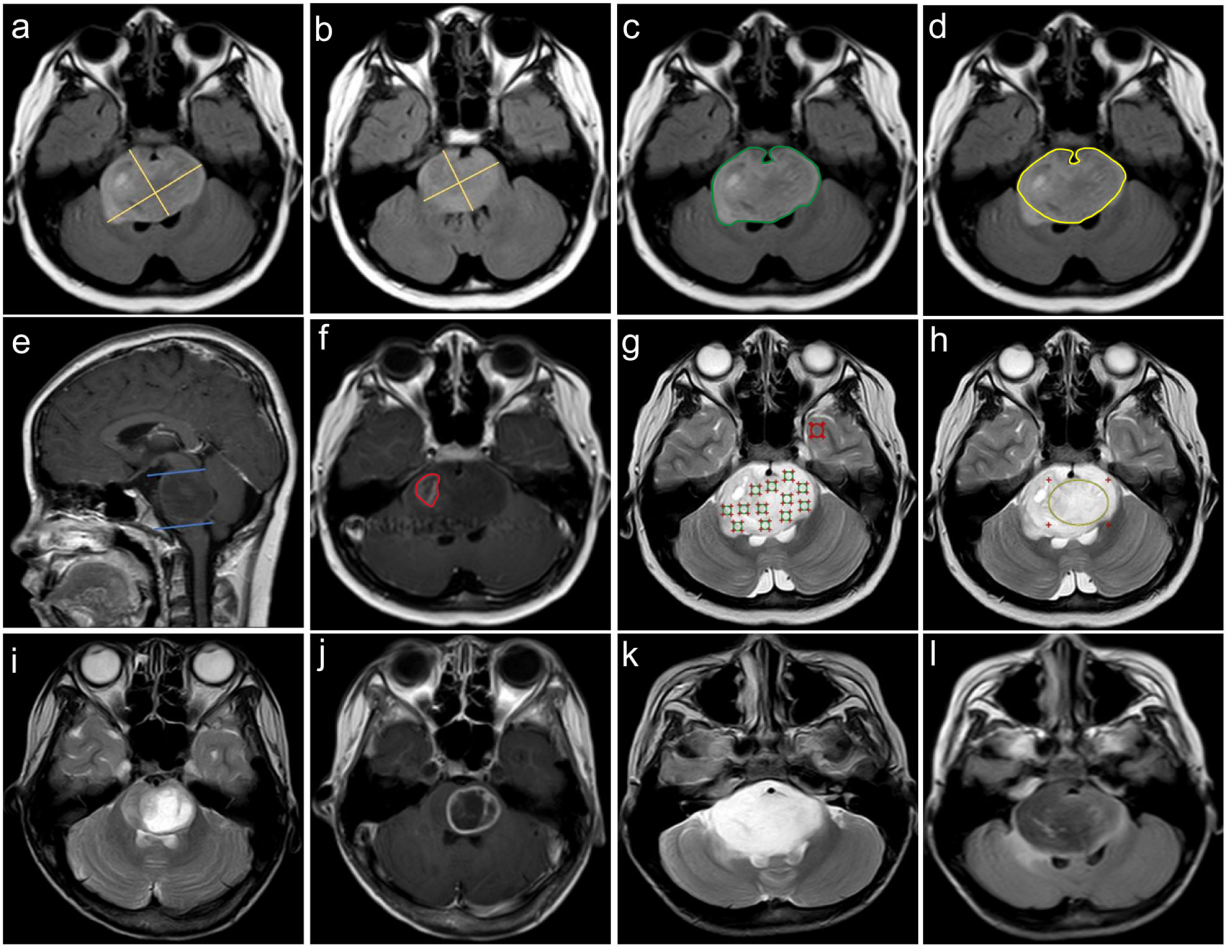
Measurement of the perpendicular diameters 2D products, T2 signal intensity, and volume (including total tumor, pons, and ring enhancement) and DIPG morphologic characteristics. **a**, **b** Measurement of 2D products of the largest perpendicular diameters before and after DIPG radiotherapy. **c**–**f** Measurement of DIPG volume: including total tumor volume (green), pontine tumor lesion volume (yellow), and ring-enhancement volume (red). **g**, **h** Minimum, and maximum T2SI ( ≥ 8 per patient; green), Mean T2SI (yellow), and normal-appearing gray matter of the temporal lobe T2SI (red). **i**, **j** An area in a tumor with clear boundaries and liquid-like signals. Contrast-enhanced T1-weighted image surrounds high signal. **k**, **l** T2-weighted hyperintense and FLAIR images showing substantially lower signal. DIPG, diffuse intrinsic pontine glioma; T2SI, T2 signal intensity

T2-weighted or FLAIR sequences can be used to measure the tumor diameter [20]. Tumor volume and 2D products of perpendicular diameters were measured using axial T2-FLAIR combined with sagittal T1WI images. MRI data were loaded using RadiAnt DICOM Viewer (Medixant; RadiAnt DICOM Viewer Software, Version 2021.1; https://www.radiantviewer.com) on June 27, 2021. Volume measurement (including total tumor, pons, and ring enhancement) was performed using 3D Slicer (http://www.slicer.org), as described by Makepeace et al [21]. For pontine tumor volume, the upper boundary was the midbrain; the lower boundary was the medulla oblongata; and the boundaries of both sides were the brachium pontis. The measurement details are shown in Fig. 1a–f. The extrapontine tumor volume was calculated as the total tumor volume minus the pontine tumor lesion volume.

### T2 signal intensity ratio

Eight or more circular region of interests (ROIs; 10–20 mm^2^) were placed on the solid component (multiple random larger layers) of the tumor. We plotted a large ROI to cover the largest tumor axial cross-section while avoiding areas of bleeding and necrosis. The minimum, mean, and maximum values of the T2 signal intensity (T2SI) were obtained. A large ROI was placed in the normal-appearing gray matter of the temporal lobe, as shown in Fig. 1g, h. T2SI_max–min_ was designated as T2SI_dif_. Finally, we normalized the T2SI_min_, T2SI_max_, T2SI_mean_, and T2SI_dif_ to the normal-appearing gray matter in the temporal lobe to obtain the relative minimum T2 signal intensity ratio (T2SR), maximum T2SR, mean T2SR, and max–min T2SR values (rT2SR_min_, rT2SR_max_, rT2SR_mean_, and rT2SR_dif_).

### Morphologic assessment

**Fig. 2:**
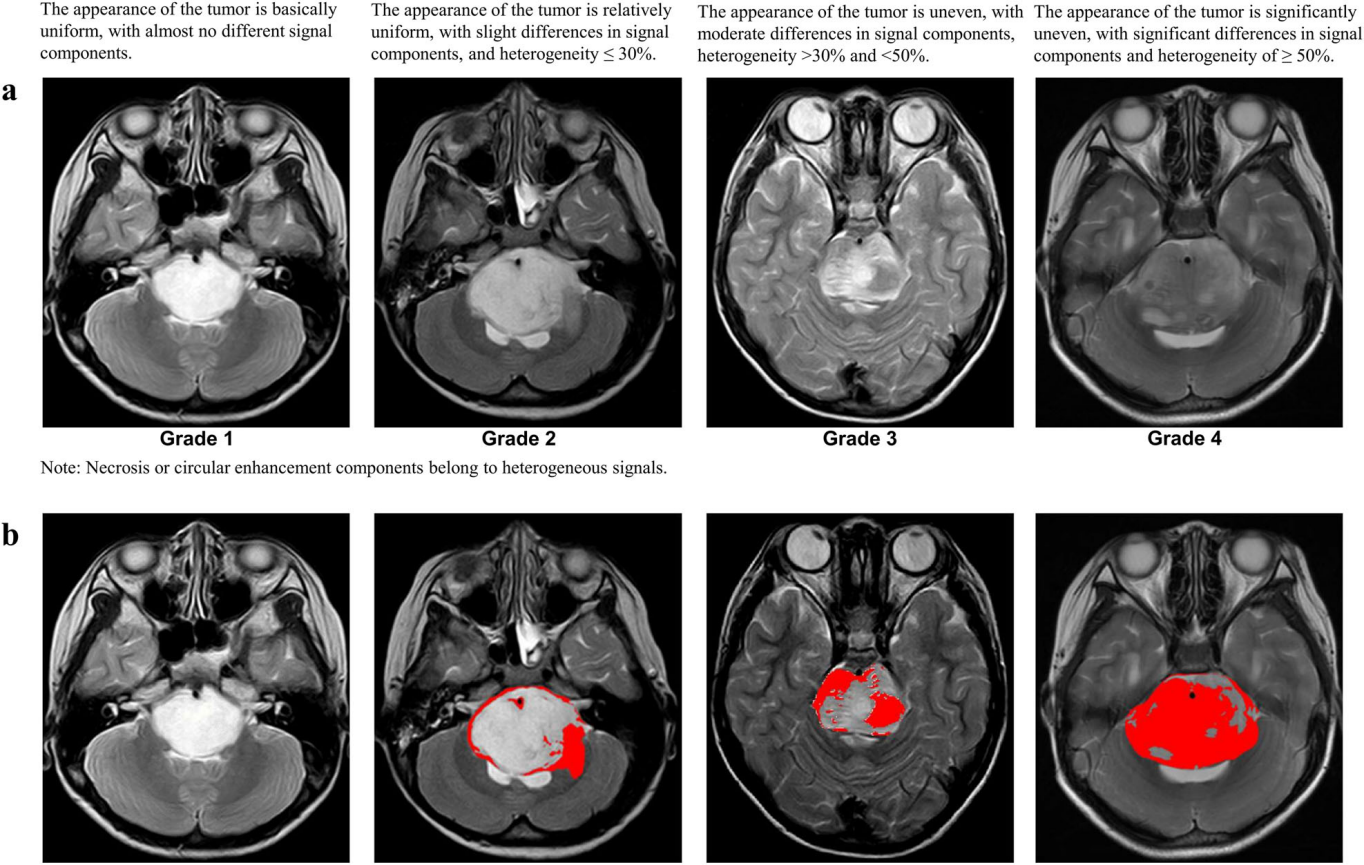
Representative images for visual grading and quantitative evaluation of heterogeneity. **a** Representative images for visual grading. **b** Quantitative evaluation of T2 signal intensity heterogeneity (Red represents the region of heterogeneity)

Ring enhancement: A contrast-enhanced T1-weighted image surrounds high signal intensity. Necrosis: An area in a tumor with clear boundaries and liquid-like signals; contrast enhancement mainly represented ring enhancement. T2-FLAIR mismatches were specified as described by Lasocki et al [22]. Examples of the different morphological features of DIPG are shown in Fig. 1i–l. We visually evaluated the heterogeneity of T2 signal intensity and divided it into four grades, as shown in Fig. 2a. To verify the visual grading of internal tumor heterogeneity, we used ImageJ software (https://imagej.net), and the grading was evaluated by adjusting the image threshold. The largest tumor axial cross-section was selected, and the heterogeneity ratio was calculated as the proportion of the area of relatively low signal intensity to the total area of the entire layer, as shown in Fig. 2b.

## MRI radiomic analysis

### Radiomic feature extraction

3D Slicer (http://www.slicer.org) was used by two observers (with seven and nine years of radiology imaging experience,) for the semi-automatic segmentation of the entire tumor area. Segmentation and subsequent feature extraction were performed using baseline MRI.

### Features extraction, selection, and model building

The radiomic features of T2-weighted and T1-enhanced images were extracted using Pyradiomics, an open-source Python package. These features included (1) first-order statistics (*n* = 252), (2) shape (*n* = 14), and (3) texture-based statistics (*n* = 1013), including gray-level co-occurrence matrix (GLCM, *n* = 336), gray-level size-zone matrix (GLSZM, *n* = 208), gray-level run-length matrix (GLRLM, *n* = 208), gray-level dependence matrix (GLDM, *n* = 196), and neighboring gray tone difference matrix (NGTDM, *n* = 65). All features were Z-score-normalized.

Features characterized by reproducibility between the outputs provided by the observers were used, whereas features with intraclass correlation coefficients (ICCs) < 0.75 were excluded. Subsequently, the least absolute shrinkage and selection operator (LASSO) algorithm was used to select the optimal features from the training set. Two models were constructed using features from the T2-weighted images (T2-weighted model) and T1-enhanced images (T1-weighted model). Finally, the radiomic scoring formula for the training set was applied to the test set to evaluate the effectiveness of the models.

### Definitions of therapeutic response

The effect of radiotherapy on tumors was determined according to the DIPG Response Assessment in Pediatric Neuro-Oncology (RAPNO) criteria [20]. Partial response (PR) was defined as a decrease ≥ 25% (compared with the baseline) in the tumor 2D products of perpendicular diameters. Progressive disease (PD) was defined as an increase ≥ 25% (compared with the baseline) in the 2D products of perpendicular diameters. The responses were classified as stable disease (SD) if they did not meet the criteria for PR or PD.

### Psuedoprogresion

Pseudoprogression was defined based on the collective criteria from recent DIPG studies [20, 23–25]; if the tumor size increased within 6 months after radiotherapy, subsequent improvement in tumor size to at least stable disease on the next MRI scan, and then resolved or stabilized without treatment, it was considered pseudoprogression. Patients who were initially assessed as PD were re-evaluated within 4–8 weeks.

### Statistical analysis

Statistical analyses were performed using SPSS (version 27.0; IBM Corp) and R software (version 4.2.0, www.R-project.org). The concordance of T2 signal intensity and quantitative parameters between the observers was examined using intraclass correlation coefficient analysis. Cohen’s kappa test was used to evaluate the observer agreement for qualitative parameters. Data that followed a normal distribution is represented as mean ± standard deviation; other data are represented as median (first and third quartiles). The Mann–Whitney U test or t-test was used to compare continuous variables between the PR and non-PR groups. Chi-square and Mann–Whitney U tests were used to compare sex, necrosis, ring enhancement, T2-FLAIR mismatch sign, and grade between the PR and non-PR groups. If there was a significant difference (*p* < 0.05) in the univariate analysis, it was included in the multivariate logistic regression for testing to predict the DIPG radiotherapy response. A nomogram was established based on the results of the multivariate analysis. Statistical significance was set at *p* < 0.05.

## Results

**Fig. 3:**
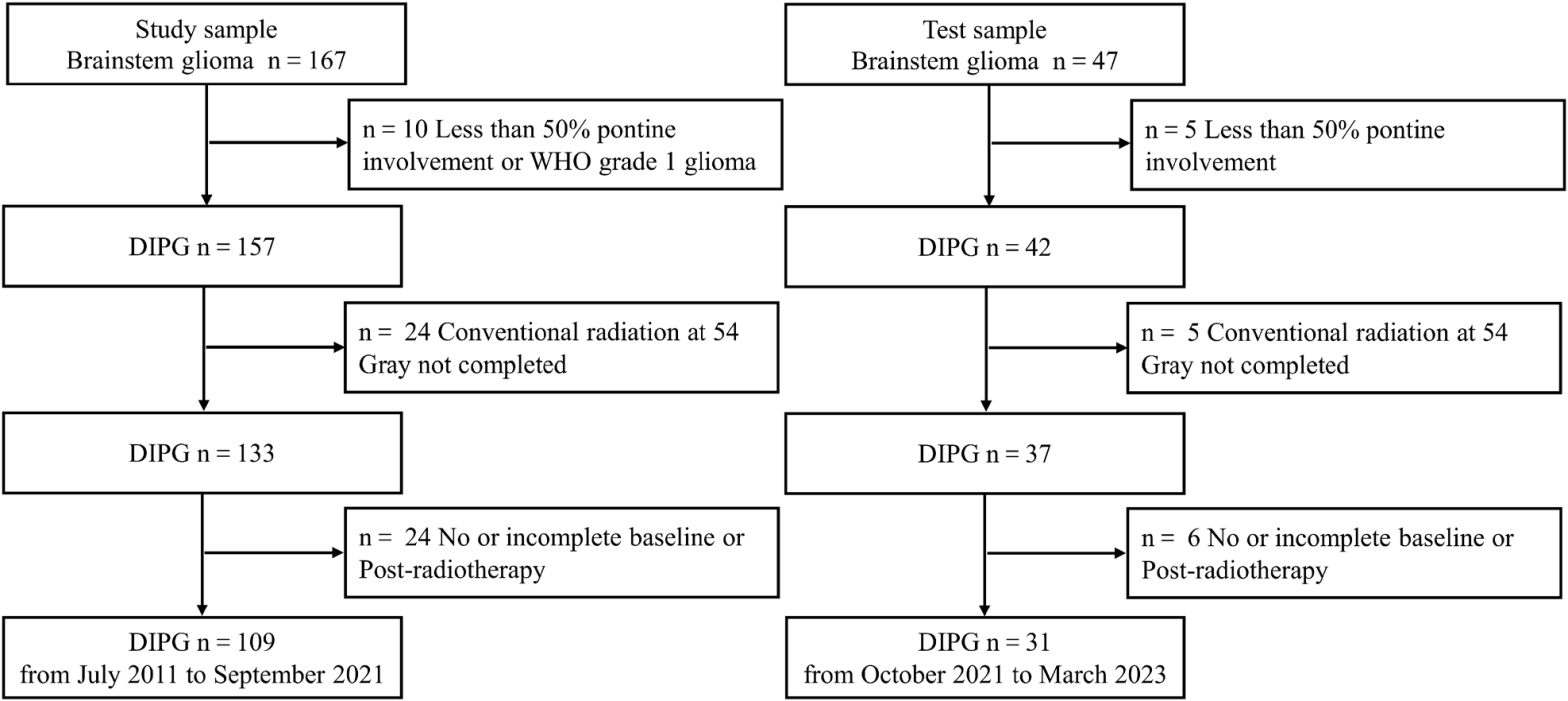
Flow diagram for patient selection. DIPG, diffuse intrinsic pontine glioma

Of 214 patients, 74 were excluded, leaving 109 and 31 patients in the training and test sets, respectively (Fig. 3). Of the remaining 140 patients, 72 (51.4%) were male and 68 (48.6%) were female, with a median age of 7 years (range, 2–18 years). The interobserver ICCs for the quantitative parameters ranged 0.89–0.98. The qualitative kappa coefficient ranged 0.76–0.97. There is substantial consistency between visual and quantitative grades for evaluating T2 heterogeneity. Details are provided in Supplementary Table 2. The clinical and imaging characteristics, grade, and radiotherapy response of the training and test sets are summarized in Table 1. According to the DIPG RAPNO criteria, the training set consisted of 109 patients with 52 showing PR, 49 showing SD, and 8 showing PD. The test set consisted of 31 patients with 12, 16, and 3 showing PR, SD, and PD, respectively. In this study, we included patients with SD and those with PD in the non-PR group. All patients were followed up until September 2023. A total of 112 patients died; 6 patients were lost to follow-up; and 22 patients survived. We compared the OS of the two groups of patients, with a median survival time of 14.0 months in the PR group, significantly higher than that of 9.0 months in the non-PR group (*p* < 0.001), as shown in Supplementary Fig. 1. We compared the changes in Karnofsky performance status scores between the PR and non-PR groups of patients before and after radiotherapy, as shown in Supplementary Fig. 2 and Supplementary Table 3. The PR ratio of T2-FLAIR mismatch but no necrosis and enhancement DIPG patients is 28/30 (93.3%), significantly higher than that of the necrosis and enhancement but without T2-FLAIR mismatch patients is 14/48 (29.2%), as shown in Supplementary Table 4.

**Table 1:** Clinical and imaging characteristics, grade, and radiotherapy response of the training and test sets

Parameter	Training set (*n* = 109)	Test set (*n* = 31)
Age (years)	2–16	3–18
Sex		
Male	54 (49.54%)	18 (58.06%)
Female	55 (50.46%)	13 (41.94%)
Tumor 2D size	20.99 ± 5.29	19.58 ± 5.60
Tumor volume	46.41 ± 16.02	43.76 ± 16.85
Necrosis	45 (41.28%)	12 (38.71%)
Ring enhancement	37 (33.94%)	12 (38.71%)
T2-FLAIR mismatch sign	23 (21.10%)	8 (25.81%)
Extra pons volume ratio	17.81 (12.25–24.75)	14.66 ± 7.27
rT2SR_min_	1.18 ± 0.22	1.19 ± 0.15
rT2SR_mean_	1.42 ± 0.25	1.45 ± 0.18
rT2SR_max_	1.60 ± 0.22	1.69 ± 0.18
rT2SR_dif_	0.43 ± 0.13	0.49 ± 0.13
Grade		
1	12 (11.01%)	1 (3.23%)
2	27 (24.77%)	11 (35.48%)
3	40 (36.70%)	16 (51.61%)
4	30 (27.52%)	3 (9.68%)
Radiotherapy response		
PR	52 (47.71%)	12 (38.71%)
SD	49 (44.95%)	16 (51.61%)
PD	8 (7.34%)	3 (9.68%)

*rT2SR*_*min*_ relative minimum T2 signal intensity ratio, *rT2SR*_*mean*_ relative mean T2 signal intensity ratio, *rT2SR*_*max*_ relative maximum T2 signal intensity ratio, *rT2SR*_*dif*_ relative (max–min) T2 signal intensity ratio

**Fig. 4:**
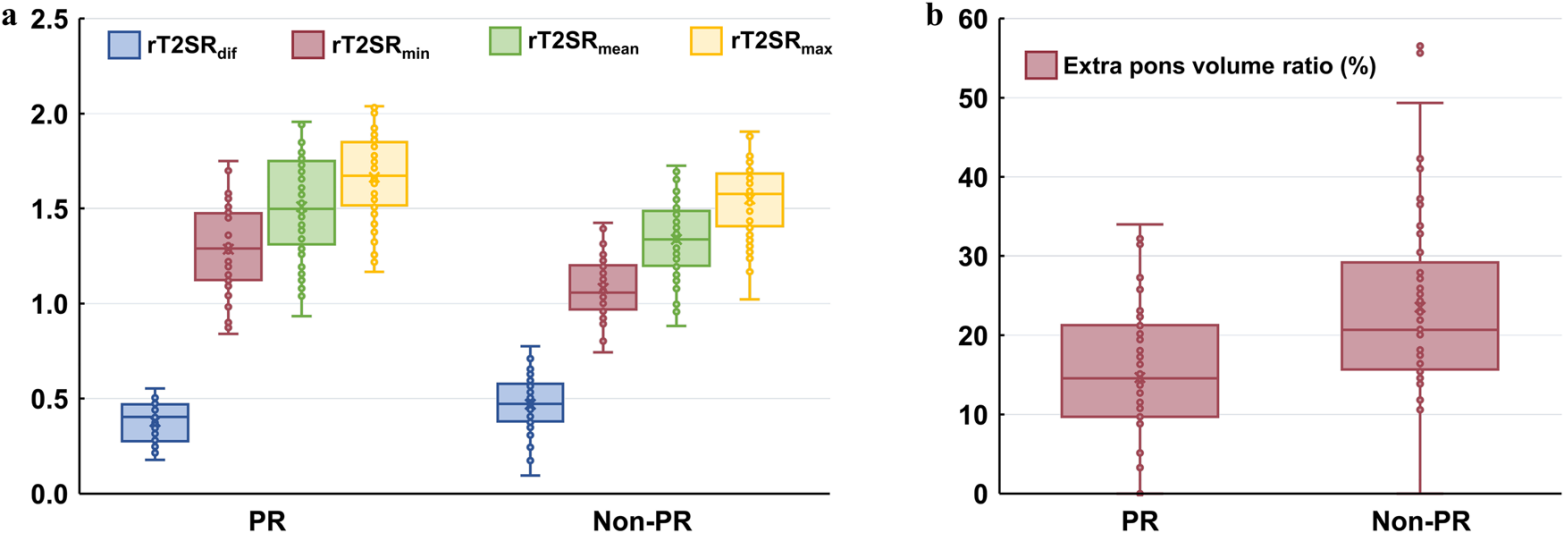
Box plots showing rT2SR_min_, rT2SR_mean_, rT2SR_max_, rT2SR_dif_, and extrapontine volume ratio in patients with PR and non-PR. **a** Boxplots of relative T2SR characteristics in patients with PR and non-PR. **b** Boxplots showing extrapontine volume ratio in patients with PR and non-PR. Boxes indicate interquartile range; lines in boxes indicate median values. The whiskers extend from the median to ± 1.5 × interquartile ranges. T2SR, T2 signal intensity ratio; rT2SR_min_, relative minimum T2 signal intensity ratio; rT2SR_mean_, relative mean T2 signal intensity ratio; rT2SR_max_, relative maximum T2 signal intensity ratio; rT2SR_dif_, relative (max–min) T2 signal intensity ratio

Univariate analyses revealed statistically significant differences (*p* < 0.05) between the two groups in the training set (necrosis, T2-FLAIR mismatch sign, extrapontine volume ratio, rT2SR_min_, rT2SR_mean_, rT2SR_max_, rT2SR_dif_, and grade), are shown in Table 2. Patients of the non-PR group showed significantly lower rT2SR_min_, rT2SR_mean_, rT2SR_max_ than those of PR group participants (*p* < 0.05). Non-PRs showed significantly higher rT2SR_dif_ values than those of PRs (*p* < 0.05). Non-PRs also showed a significantly higher extra pons volume ratio than those of PRs (*p* < 0.05). The rT2SR_min_, rT2SR_mean_, rT2SR_max_, rT2SR_dif_, and extrapontine volume ratios in patients with and without PR are shown in Fig. 4. The AUC and cut-off values for quantitative parameters in the training set that distinguished between the PR and non-PR groups are shown in Table 3.

**Table 2:** Univariate and multivariate logistic regression results for the prediction of pediatric DIPG radiotherapy response in the training set

Parameter	Training set (*n* = 109)		*p* value
**Univariate**	**PR (*n*** = **52)**	**Non-PR (*n*** = **57)**	
Age (years)	6.50 (4.25–8.75)	8.00 (6.00–9.00)	0.063
Sex			0.635
Male	27	27	
Female	25	30	
Tumor 2D size	20.24 ± 4.91	21.68 ± 5.57	0.155
Tumor volume	43.58 ± 14.66	48.99 ± 16.88	0.078
Necrosis			< 0.001^***^
Present	13	32	
Absent	39	25	
Ring enhancement			0.060
Present	13	24	
Absent	39	33	
T2-FLAIR mismatch sign			< 0.001^***^
Present	20	3	
Absent	32	54	
Extra pons volume ratio	14.55 (9.70–21.28)	20.69 (15.67–29.18)	< 0.001^***^
rT2SR_min_	1.286 ± 0.230	1.080 ± 0.145	< 0.001^***^
rT2SR_mean_	1.509 ± 0.264	1.337 ± 0.203	< 0.001^***^
rT2SR_max_	1.6647 ± 0.231	1.549 ± 0.189	0.005^**^
rT2SR_dif_	0.379 ± 0.104	0.469 ± 0.137	< 0.001^***^
Grade			< 0.001^***^
1	11	1	
2	21	6	
3	16	24	
4	4	26	
**Multivariate**			
Extra pons volume ratio			0.003^**^
rT2SR_dif_			0.015^*^
Grade			< 0.001^***^
1			
2			
3			
4			

^*^ *p* < 0.05, ^**^ *p* < 0.01, ^***^ *p* < 0.001

*DIPG* diffuse intrinsic pontine glioma, *rT2SR*_*min*_ relative minimum T2 signal intensity ratio, *rT2SR*_*mean*_ relative mean T2 signal intensity ratio, *rT2SR*_*max*_ relative maximum T2 signal intensity ratio, *rT2SR*_*dif*_ relative (max–min) T2 signal intensity ratio

**Table 3:** Diagnostic accuracy of univariate quantitative parameters for differentiating pediatric DIPG radiotherapy response in the training set

Parameters	AUC (95% CI)	Sensitivity (%)	Specificity (%)	Cut-off value
rT2SR_min_	0.760 (0.669, 0.837)	53.85	92.98	1.268
rT2SR_mean_	0.696 (0.601, 0.780)	63.46	71.93	1.454
rT2SR_max_	0.658 (0.561, 0.746)	48.08	82.46	1.712
rT2SR_dif_	0.692 (0.597, 0.777)	80.77	49.12	0.486
Extra pons volume ratio	0.721 (0.627, 0.803)	38.46	94.74	11.524

*DIPG* diffuse intrinsic pontine glioma, *CI* confidence interval, *rT2SR*_*min*_ relative minimum T2 signal intensity ratio, *rT2SR*_*mean*_ relative mean T2 signal intensity ratio, *rT2SR*_*max*_ relative maximum T2 signal intensity ratio, *rT2SR*_*dif*_ relative (max–min) T2 signal intensity ratio

**Fig. 5:**
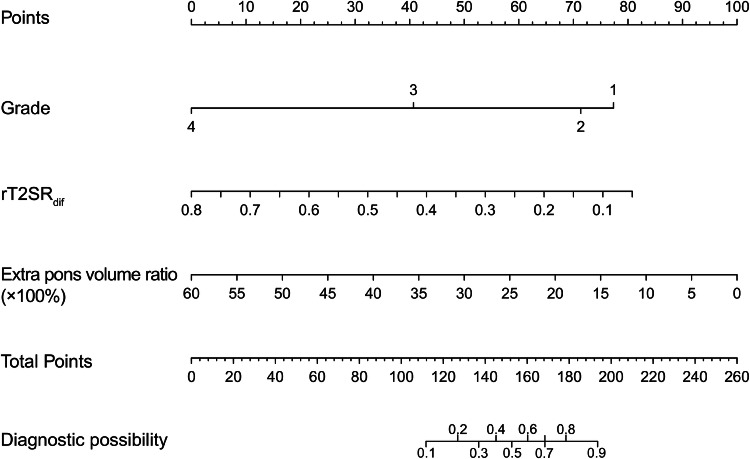
Nomogram constructed based on the combined model. Each point that corresponds to specific variable is on the uppermost point scale. The sum of all points is the total points. The point total projected at the bottom scale indicates the probability of PR in patients with DIPG. DIPG, diffuse intrinsic pontine glioma; rT2SR_dif_, relative (max–min) T2 signal intensity ratio

**Fig. 6:**
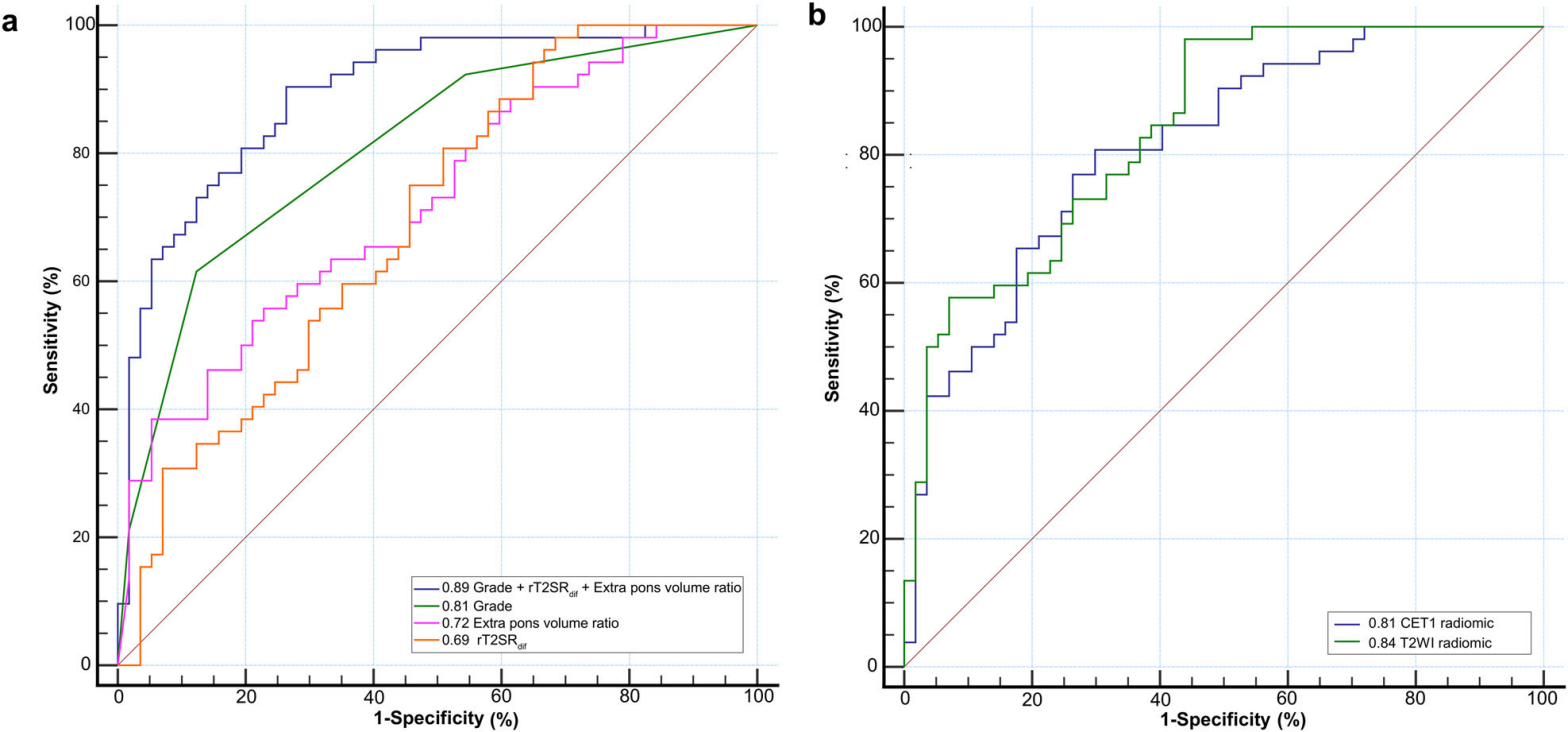
Receiver operating characteristic curves for single-parameter, three-parameter, and radiomic models were compared to predict DIPG radiotherapy response. **a** Receiver operating characteristic curves for single-parameter, three-parameter, were compared to predict DIPG radiotherapy response. **b** Receiver operating characteristic curves for radiomic models were compared to predict DIPG radiotherapy response. DIPG, diffuse intrinsic pontine glioma; rT2SR_dif_, relative (max–min) T2 signal intensity ratio; CET1, contrast-enhanced T1WI

The multivariate regression results are shown in Table 2. A nomogram was established based on the multivariate regression results, as shown in Fig. 5. The best-performing model used in the training group to predict the DIPG radiotherapy response consisted of three variables (extra pons volume ratio, rT2SR_dif_, and grade). The AUC of the three-variable model (extra pons volume ratio, rT2SR_dif_, and grade) was 0.89, the sensitivity was 90.38%, and the specificity was 73.68%, as shown in Fig. 6a. Using the test set, the AUC of the DIPG radiotherapy response prediction model was 0.91 (sensitivity and specificity of 91.67% and 84.21%, respectively) as shown in Table 4.

**Table 4:** AUC values of multiparameter models and radiomic models in the training and test sets

	AUC (95% CI)	Sensitivity (%)	Specificity (%)
**Train set**			
Extra pons volume ratio, rT2SR_dif_, and Grade	0.893 (0.819, 0.944)	90.38	73.68
T2WI radiomic model	0.840 (0.758, 0.903)	98.08	56.14
CET1 radiomic model	0.813 (0.727, 0.881)	80.77	70.18
**Test set**			
Extra pons volume ratio, rT2SR_dif_, and Grade	0.912 (0.754, 0.983)	91.67	84.21
T2WI radiomic model	0.825 (0.646, 0.937)	75.00	89.47
CET1 radiomic model	0.811 (0.631, 0.929)	100.00	57.89

*AUC* area under the curve, *CI* confidence interval, *rT2SR*_*dif*_ relative (max–min) T2 signal intensity ratio, *CET1* T1WI-enhanced

### MRI radiomic analysis

In the final feature selection using the LASSO method, five and six radiomic features were included in the T2-weighted and T1-enhanced images, respectively. Details are provided in Supplementary Table 5. Using the training set, the AUCs of DIPG radiotherapy response prediction models were 0.84 (sensitivity and specificity were 98.08% and 56.14%, respectively) for the T2-weighted model and 0.81 (sensitivity and specificity were 80.77% and 70.18%, respectively) for the T1-enhanced model as shown in Fig. 6b and Table 4. Using the test set, the AUCs for DIPG radiotherapy response prediction models were 0.83 (sensitivity and specificity of 75.00% and 89.47%, respectively) for the T2-weighted model and 0.81 (sensitivity and specificity were 100.00% and 57.89%, respectively) for the T1-enhanced model. Further explore the correlation between radiomic features and overall survival, as shown in Supplementary 6.

## Discussion

There is an increasing need to develop a reliable diagnostic method to accurately predict the radiotherapy response in patients with DIPG. In this study, we developed a predictive model that combines T2-weighted (quantitative and qualitative) image features with a quantitative assessment of extrapontine extension. In our prediction model (the combination of T2-weighted quantification, qualitative analysis, and extra pons volume ratio), the AUC on the training set is 0.89, and it shows good discrimination in the independent test set (AUC, 0.91). The AUCs of T2-weighted and T1-enhanced radiomics prediction models on the training set were 0.84 and 0.81, respectively. They showed good distinguishability on the independent test set, with AUCs of 0.83 and 0.81, respectively. Glioma is a heterogeneous disease with intratumoral heterogeneity at both the genetic and histopathological levels, especially in high-grade gliomas [26–28]. T2-weighted images are superior to other MR image types in determining DIPG intratumoral heterogeneity [19].

We used visual grading to qualitatively analyze T2-weighted heterogeneity. To ensure favorable memory and suitable applications, we divided the ratings into four grades, and we believe that the advantages of this visual rating are that it is extremely simple and intuitive. Therefore, this method is easier to apply than others. According to our results, the T2 signal intensity visual grading showed good interobserver consistency. Moreover, while subjectively evaluating the T2 signal intensity, we used a quantitative method by multiple observers to measure the T2 signal intensity ratio of the tumor area relative to the normal gray matter of the temporal lobe. This repeatable evaluation method is conducive to further comparison of research results between different field strengths.

Non-PRs showed significantly lower rT2SR_min_, rT2SR_mean_, and rT2SR_max_ than that of PRs. Non-PRs also showed significantly higher rT2SR_dif_ values than that of PRs. DIPGs are typically hyperintense on T2-weighted MRI sequences [20]. Areas with lower T2 signal intensities may have a greater likelihood of diffusion limitation and enhancement [29, 30]. In addition, DIPG studies have shown that areas of signal hypointensity on T2-weighted images correspond to the foci of anaplasia and hypercellularity [29, 31]. We quantified differences in rT2SR values to evaluate tumor heterogeneity. Therefore, regions displaying high T2 signal intensity ratio differences may reflect highly heterogeneous regions with spatial changes in cellular structure and various pathological components.

DIPGs located in an eloquent brain region preclude surgical resection; research shows the safety of DIPG biopsy [32] in providing data for treatment decisions; however, the sample size obtained from stereotactic biopsy is relatively small and cannot completely decipher the overall heterogeneity of the tumor. Extrapontine extension is an unfavorable prognostic factor in patients with DIPG [1, 21]. We discovered a method for measuring extrapontine extension in previous studies [21], which may be more accurate.

Those with T2-FLAIR mismatch sign have a good radiotherapy response [16], and the same results were obtained in the univariate analysis. However, the T2-FLAIR mismatch sign was mostly characterized as grade 1 or grade 2 in the visual evaluation method of T2 signal intensity heterogeneity used in this study. Hence, T2-FLAIR mismatch sign was not included in the multivariate analysis. Moreover, radiomic models can be used to predict the prognosis of patients with DIPG [33–35]. However, few studies have focused on radiomic models and DIPG radiotherapy responses. Radiomics has the advantage of providing better disease representation by revealing high-dimensional features and subpattern changes beyond visual assessment [36]. The use of Radiomic models in this study to predict DIPG radiotherapy response also demonstrated good discrimination.

In the present study, we confirmed that the survival period in the PR group was significantly longer than that in the non-PR group. Further analysis was conducted on the changes in KPS scores of the two groups of patients before and after radiotherapy; the PR group had a better quality of life. Assuming that the overall survival of patients cannot be significantly improved in a short period, the quality of life of these patients will become equally important.

This study has some limitations. This was a retrospective, single-center study. Further prospective multicenter studies are required to validate our results. There are few patients undergoing biopsy, and only a small portion of patients have genetic data. Accordingly, we will incorporate genetic data to improve the diagnostic ability of the model in future research.

## Conclusion

Combining T2-weighted quantification with qualitative and extrapontine volume ratios can accurately predict DIPG radiotherapy response. Early identification of radiotherapy response to DIPG is helpful for personalized treatment and prognostic assessment for patients with DIPG.

## Supplementary information

Supplementary Material

## Abbreviations

DIPG: Diffuse intrinsic pontine glioma
FLAIR: Fluid-attenuated inversion recovery
ICC: Intraclass correlation coefficient
LASSO: Least absolute shrinkage and selection operator
OS: Overall survival
PD: Progressive disease
PR: Partial response
RAPNO: Response Assessment in Pediatric Neuro-Oncology
ROI: Region of interest
rT2SR_dif_: Relative (max–min) T2 signal intensity ratio
rT2SR_max_: Relative maximum T2 signal intensity ratio
rT2SR_mean_: Relative mean T2 signal intensity ratio
rT2SR_min_: Relative minimum T2 signal intensity ratio
SD: Stable disease
T2SI: T2 signal intensity
T2SI_dif_: (max–min) T2 signal intensity
T2SI_max_: Maximum T2 signal intensity
T2SI_mean_: Mean T2 signal intensity
T2SI_min_: Minimum T2 signal intensity
T2SR: T2 Signal intensity ratio
