# Supplementary material for: Pediatric diffuse intrinsic pontine glioma radiotherapy response prediction: MRI morphology and T2 intensity-based quantitative analyses
Source: Eur Radiol. 2024 Jun 21;34(12):7962–72. doi: 10.1007/s00330-024-10855-9 (PMC11557687; doi:10.1007/s00330-024-10855-9)
Supplement: Supplementary file 1 — Supplementary Material [file 330_2024_10855_MOESM1_ESM.pdf]

Pediatric diffuse intrinsic pontine glioma radiotherapy response prediction: MRI morphology and T2 intensity-based quantitative analyses

Electronic Supplementary Material (ESM)

Eur Radiol (2024) Yu X, Li S, Mai W et al.

Supplementary Figure 1. Kaplan-Meier survival analysis results of radiotherapy response in patients with DIPG.

DIPG, diffuse intrinsic pontine gliomas

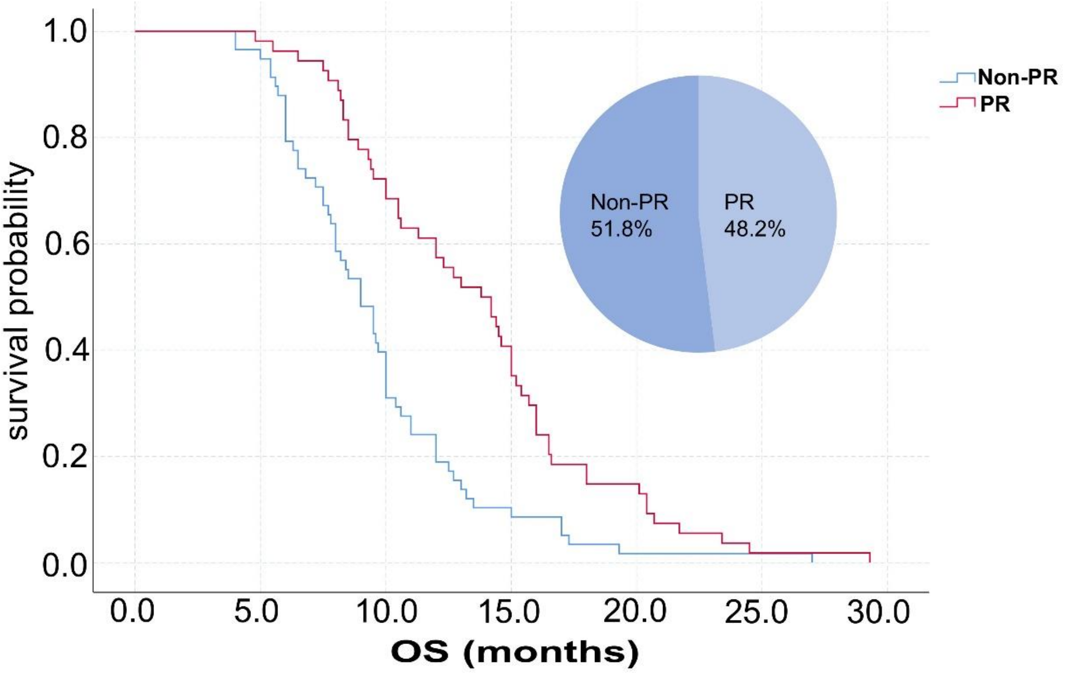

Supplementary Figure 2. Changes in Karnofsky score of patients with DIPG before and after radiotherapy

DIPG, diffuse intrinsic pontine glioma; KPS, Karnofsky Performance Status

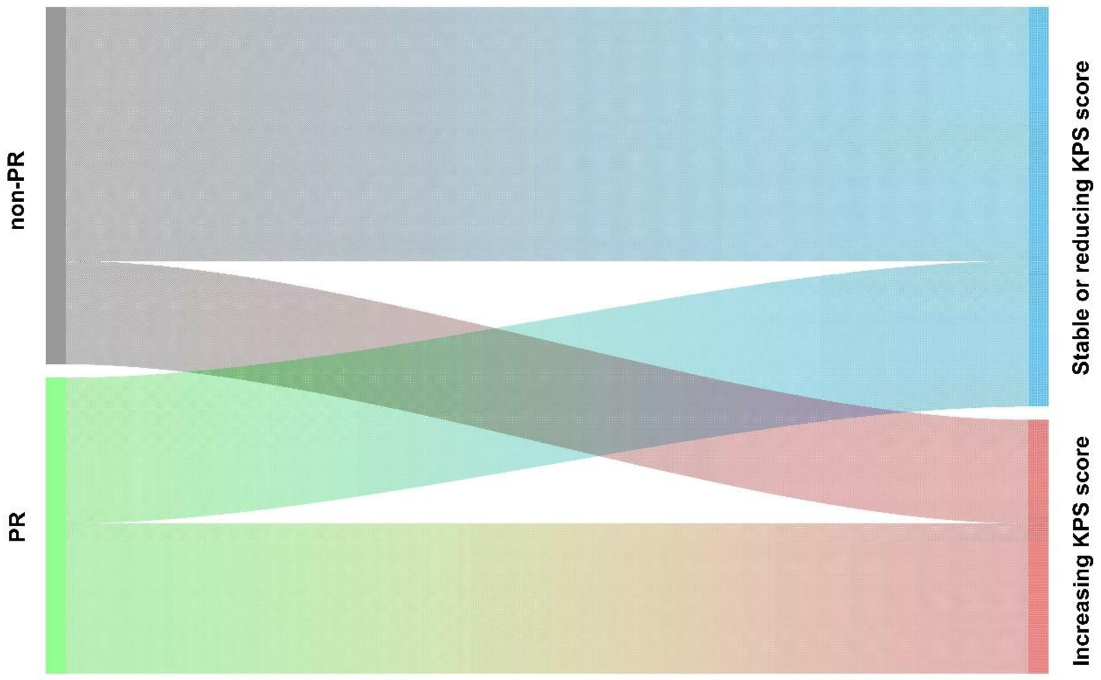

Supplementary Table 1. MRI Parameters

Sequence	TE* (ms)	TR* (ms)	Slice thickness (mm)	Interslice spacing (mm)
T1WI	15–28	488–1900	5/5.5	0/1
T2WI	110–120	3980–4480	5/5.5	0/1
T2WI-FLAIR	120–135	6000–9480	5/5.5	0/1

Abbreviations: FLAIR, fluid-attenuated inversion recovery; TE, echo time; TR, repetition time.

Supplementary Table 2.1. Interobserver repeatability of DIPG quantitative parameters

**Measurement**	**ICC (95% CI)**
Tumor cross product (cm^2)	0.918 (0.898, 0.935)
Tumor volume (cm^3)	0.966 (0.953, 0.976)
Pontine lesion volume (cm^3)	0.951 (0.931, 0.965)
Ring enhancement volume (cm^3)	0.980 (0.965, 0.989)
$T2SI_{min}$	0.912 (0.879, 0.936)
$T2SI_{mean}$	0.936 (0.911, 0.953)
$T2SI_{max}$	0.892 (0.852, 0.921)
$T2SI_{temporal\ lobe}$	0.917 (0.886, 0.940)

Abbreviations: DIPG, diffuse intrinsic pontine glioma; ICC, intraclass correlation coefficient; CI, confidence interval; $T2SI_{min}$, minimum T2 signal intensity; $T2SI_{mean}$, mean T2 signal intensity; $T2SI_{max}$, maximum T2 signal intensity; $T2SI_{temporal\ lobe}$, temporal lobe T2 signal intensity

Supplementary Table 2.2. Interobserver repeatability of DIPG qualitative parameters

**Parameter**	**Kappa coefficient (95% CI)**
Necrosis	0.940 (0.869, 0.986)
Ring enhancement	0.969 (0.920, 1.000)
T2-FLAIR mismatch sign	0.879 (0.775, 0.962)
Grade	0.760 (0.668, 0.844)
Visual and quantitative grade	0.800 (0.712, 0.880)

Abbreviations: DIPG, diffuse intrinsic pontine glioma; CI, confidence interval

Supplementary Table 3. Changes in the KPS scores of patients PR and non-PR after radiotherapy

	**Increasing KPS score**	**Stable or reducing KPS score**	***P* value**
**Radiotherapy response**			0.006^{**}
PR	33	31	
Non-PR	22	54	

* $P < 0.05$, ** $P < 0.01$.

Supplementary Table 4. Compared the differences in PR between T2-FLAIR mismatch but no necrosis and enhancement and necrosis and enhancement but without T2-FLAIR mismatch.

	**PR**	**Non-PR**	***P* value**
**Characteristic**			$< 0.001^{***}$
T2-FLAIR mismatch and no necrosis and enhancement	28	2	
Necrosis and enhancement but without T2-FLAIR mismatch	14	34	

* $P < 0.05$, ** $P < 0.01$, *** $P < 0.001$.

Supplementary Table 5. Radiomic features and weights.

T2-weighted model

Feature	Weight
log-sigma-2-0-mm-3D_glszm_ZoneVariance	0.25742
wavelet-LLL_gldm_DependenceVariance	0.20068
wavelet-LHL_gldm_LargeDependenceLowGrayLevelEmphasis	-0.19914
wavelet-HLH_glcm_SumEntropy	-0.18187
wavelet-LLL_firstorder_RootMeanSquared	0.10332

T1-enhanced model

Feature	Weight
wavelet-HHH_glcm_ClusterShade	-1.75586
wavelet-HHH_glrlm_RunLengthNonUniformity	-0.64088
lbp-3D-m1_glcm_Idm	-0.44618
wavelet-HHH_glcm_Contrast	0.439437
wavelet-LHL_glrlm_LongRunLowGrayLevelEmphasis	-0.38319
log-sigma-3-0-mm-3D_glszm_GrayLevelNonUniformity	0.332178

Supplementary 6 Radiomic features, weights, and overall survival.

LASSO method showed no correlation between T2WI radiomic features and overall survival.

Supplementary Table 6.1 T1-enhanced radiomic features and weights.

Feature	Weight
diagnostics_Image.original_Size	-0.370675
wavelet-LHH_glcm_DifferenceAverage	-1.229477
wavelet-HLH_gldm_HighGrayLevelEmphasis	-0.302951
wavelet-HHH_glcm_Contrast	1.165232

Supplementary Table 6.2 Multivariate analyses of the T1-enhanced radiomic features with overall survival

**Multivariate**	**HR (95% CI)**	***P* value**
diagnostics_Image.original_Size	0.69 (0.53, 0.91)	0.008^{**}
wavelet-LHH_glcm_DifferenceAverage	0.29 (0.11, 0.76)	0.012^{*}
Wavelet-HLH_gldm_HighGrayLevelEmphasis	0.74 (0.59, 0.92)	0.008^{**}
Wavelet-HHH_glcm_Contrast	3.21 (1.02, 10.04)	0.045^{*}

* P < 0.05, ** P < 0.01, *** P < 0.001.

Abbreviations: CI, confidence interval
